# Morphological and Phagocytic Profile of Microglia in the Developing Rat Cerebellum^1^,^2^,^3^

**DOI:** 10.1523/ENEURO.0036-15.2015

**Published:** 2015-08-31

**Authors:** Miguel Perez-Pouchoulen, Jonathan W. VanRyzin, Margaret M. McCarthy

**Affiliations:** 1 Department of Pharmacology, University of Maryland School of Medicine, Baltimore, Maryland 21201; 2 Program in Neuroscience, University of Maryland School of Medicine, Baltimore, Maryland 21201

**Keywords:** cell death, development, microglia, phagocytosis, sex difference, vermis

## Abstract

Microglia are being increasingly recognized as playing important roles in neurodevelopment. The cerebellum matures postnatally, undergoing major growth, but the role of microglia in the developing cerebellum is not well understood. Using the laboratory rat we quantified and morphologically categorized microglia throughout the vermis and across development using a design-based unbiased stereology method. We found that microglial morphology changed from amoeboid to ramified during the first 3 postnatal weeks in a region specific manner. These morphological changes were accompanied by the sudden appearance of phagocytic cups during the third postnatal week from P17 to P19, with an approximately fourfold increase compared with the first week, followed by a prompt decline at the end of the third week. The microglial phagocytic cups were significantly higher in the granular layer (∼69%) than in the molecular layer (ML; ∼31%) during a 3 d window, and present on ∼67% of microglia with thick processes and ∼33% of microglia with thin processes. Similar proportions of phagocytic cups associated to microglia with either thick or thin processes were found in the ML. We observed cell nuclei fragmentation and cleaved caspase-3 expression within some microglial phagocytic cups, presumably from dying granule neurons. At P17 males showed an approximately twofold increase in microglia with thin processes compared with females. Our findings indicate a continuous process of microglial maturation and a nonuniform distribution of microglia in the cerebellar cortex that implicates microglia as an important cellular component of the developing cerebellum.

## Significance Statement

Microglia are the resident immune cells of the brain and constantly survey their local environment in order to eliminate cellular debris after injury or infection. During brain development, microglia participate in neurite growth, synaptic pruning, and apoptosis, all of which are essential processes to the establishment of neuronal circuits. The cerebellum undergoes major growth and synaptic reorganization after birth, leading to the development of cerebellar circuits which are involved in motor and cognitive functions. The role of microglia in the developing cerebellum is not well understood. This study provides important foundational profiles of microglial development in the cerebellum, a vulnerable structure to alteration during development, and contributes to the growing appreciation of the clearance activity of microglia during postnatal development.

## Introduction

Microglia are the resident macrophages of the CNS and play important roles during both normal functioning and in disease or injury. Microglia exhibit diverse morphological features across the CNS and phases of the lifespan (Tremblay et al., 2011). In the adult brain, microglia are known to actively survey their environment through their ramified processes and they dramatically change their morphology in response to damage or infection in order to repair the CNS (Ayoub and Salm, 2003; Nimmerjahn et al., 2005; Ransohoff and Perry, 2009; Nayak et al., 2014). These morphological changes are accompanied by phagocytosis to remove dead cells or cellular debris (Vargas et al., 2005; Cǎtǎlin et al., 2013), giving microglia the title of the scavengers of the CNS. Recent evidence suggests microglia are involved in normal development of the brain including neurite growth, synaptic pruning, spinogenesis, and apoptosis (Marín-Teva et al., 2004; Paolicelli et al., 2011; Schafer et al., 2012; Lenz et al., 2013; Kaur et al., 2014). During development microglia undergo morphological changes in both cell body and configuration of their processes, changing from round to ramified, with intermediate stages as the brain matures (Wu et al., 1992; Schwarz et al., 2012). Thus, microglia have important functions impacting the development and formation of neural circuits in the CNS. What is not well understood is whether these functions occur according to a developmental timeframe and how they might differ between brain regions.

The cerebellum is a brain structure involved in many functions including motor control and coordination (Glickstein, 1992; Glickstein et al., 2009), as well as nonmotor functions, such as attention, working memory, language, nociception, pain, addiction, and reward (Rapoport et al., 2000; Gottwald et al., 2003; Holstege et al., 2003; Saab and Willis, 2003; Miquel et al., 2009; Strick et al., 2009; Durisko and Fiez, 2010; Moulton et al., 2010, 2014; Murdoch, 2010; Strata, 2015). The anatomy of the cerebellum consists of an organized and uniform cytoarchitecture that allows systematic and efficient communication among the cerebellar neurons (Voogd and Glickstein, 1998; Sillitoe and Joyner, 2007; Apps and Hawkes, 2009). The human cerebellum matures postnatally and undergoes major growth and neuronal reorganization during the first 2 years after birth (Abraham et al., 2001; ten Donkelaar et al., 2003; Butts et al., 2014). In rats, cerebellar maturation occurs during the first 3 postnatal weeks with dramatic anatomical changes involving an increase in both cell density and mass volume (Heinsen, 1977; Altman, 1982; Goldowitz and Hamre, 1998). The cerebellar cortex consists of three anatomical layers containing different types of neurons with distinct timeframes of maturation. Maturation of the cerebellar circuitry involves the production and removal of cells, as well as spinogenesis and synaptogenesis, among others (Altman, 1972; Wood et al., 1993; Sarna and Hawkes, 2003; Tanaka, 2009; Haraguchi et al., 2012). Microglia regulate synapses in the developing brain in areas, such as the visual cortex, hippocampus, and retinogeniculate system (Tremblay et al., 2010; Paolicelli et al., 2011; Schafer et al., 2012). However, the number of studies on the role of microglia during postnatal development remain relatively low. In the cerebellum microglia are distributed in both gray and white matter throughout the lifespan across diverse species, and there is a distinct arrangement of microglia processes according to location in the cerebellar cortex (Ashwell, 1990; Vela et al., 1995; Cuadros et al., 1997). Microglia can induce apoptosis of Purkinje neurons *in vitro* (Marín-Teva et al., 2004), but this is not established *in vivo* under normal conditions and overall the role of microglia during postnatal development of the cerebellum is not well understood.

The overarching goal of this report is to profile anatomical changes of microglia during postnatal development of the cerebellum. We hypothesized microglia change their morphological profile and cell density in direct relationship to the maturation of the cerebellum, as well as the anatomical location within the cerebellar cortex. Thus, the purpose of the current study was twofold: first, to identify the morphological profile of microglia across the postnatal developing cerebellum using the ionized calcium-binding adapter molecule 1 (Iba1), which is a microglia marker (Ito et al., 1998); and second, to determine whether the morphological profile of microglia, as well as their phagocytic capability, differ according to location in the cerebellar cortex.

## Materials and Methods

### Animals

Timed pregnant Sprague-Dawley rats purchased from Charles River Laboratories or raised in our breeding colony were allowed to deliver naturally under standard laboratory conditions. Male and female rat pups were used and the day of birth was denoted as postnatal day (P) 0. All animals were housed in polycarbonate cages (20 × 40 × 20 cm) with corncob bedding under 12 h reverse light/dark cycle, with *ad libitum* water and food. All animal procedures were performed in accordance with the University of Maryland animal care and use committee’s regulations.

### Immunohistochemistry

Animals were deeply anesthetized with Fatal Plus (Vortech Pharmaceuticals) and transcardially perfused with 0.9% saline solution followed by 4% paraformaldehyde. The entire cerebellums were removed and postfixed overnight in 4% paraformaldehyde, and cryoprotected with 30% sucrose until they were saturated. The cerebellums were sagittally sectioned on a cryostat at a thickness of 45 µm. Free-floating cerebellar slices from different time points between P5 and P21 were rinsed with 0.1 m phosphate buffered saline (PBS), incubated with 3% hydrogen peroxide in PBS for 30 min, and then rinsed again. Sections were coincubated with a polyclonal antibody against Iba1 (1:10000, Wako Chemicals), a microglia marker (Ito et al., 1998), in 10% of bovine serum albumin (BSA) in PBS with 0.4% Triton X-100 (PBS-T) for 30 min at room temperature (RT) with constant agitation, then kept for 24 h at 4°C with constant agitation. Subsequently, sections were rinsed in PBS and incubated with biotinylated anti-rabbit secondary (1:500, Vector Laboratories) in 0.4% PBS-T for 1 h at RT followed by rinses in PBS. Sections were incubated with ABC complex (1:500, Vector Laboratories) in 0.4% PBS-T for 1 h at RT. Iba1-positive cells were visualized using nickel-enhanced diaminobenzidine (Sigma-Aldrich D-5905) as chromogen for 8–10 min incubation at which point sections displayed a dark purple staining. Finally, sections were exhaustively rinsed in PBS, mounted on silane-coated slides, cleared with ascending alcohol concentrations, defatted with xylene, and coverslipped with DPX mounting medium.

### Fluorescence immunohistochemistry

In order to colocalize microglial phagocytic cups and fragmented nuclei, Iba1 and DAPI were used, respectively. Free-floating tissue sections from P17 cerebellums were rinsed with 0.1 m PBS, incubated with 3% hydrogen peroxide in PBS for 30 min, rinsed, and then incubated with 0.3 m glycine in 0.4% PBS-T for 60 min. Subsequently, sections were incubated with Iba1 (1:1000, Wako Chemicals) in 0.4% PBS-T containing 10% BSA for 30 min at RT with constant agitation, and then kept for 24 h at 4°C with constant agitation. After primary incubation, sections were rinsed in PBS and incubated with the secondary antibody anti-rabbit AlexaFluor 594 (1:500; Invitrogen) in PBS-T for 120 min in the dark. Sections were then rinsed, mounted and cover-slipped using Hardset mounting medium containing DAPI (Vector Laboratories).

To colocalize dead or dying cells with microglial phagocytic cups, we followed the fluorescence protocol described above to identify the cellular death marker cleaved caspase-3 (1:750, Cell Signaling Technology) and Iba1 (1:1000, Abcam) on P17 cerebellar sections (both cleaved caspase-3 and Iba1 antibodies were incubated together). Anti-rabbit AlexaFluor 488 (1:500; Invitrogen) and anti-goat AlexaFluor 594 (1:500, Invitrogen) were used as secondary antibodies.

### Nissl staining

Sagittal sections (45 µm) from P5, P7, P14, P17, and P21 vermis were stained with cresyl violet in order to identify pyknotic bodies. Cerebellar sections were washed with PBS 0.1 m, mounted and dried for 24 h. Subsequently, sections were hydrated with a series of decreasing concentrations of ethanol (95, 70, and 50%) for 2 min followed by two washes of distilled water (dH_2_O) for 1 min. After a 30 sec incubation in 0.1% cresyl violet, sections were washed with dH_2_O for 1 min and, then incubated in 70% ethanol for 2 min before the differentiation step in 5% alcohol acid (95% ethanol + 5% acetic acid) for 5 min. Sections were then dehydrated with two washes of 95% ethanol for 2 and 1 min, respectively, and a final incubation in xylene for 3 min before cover-slipping with DPX.

### Stereological counts

**Figure 1.**
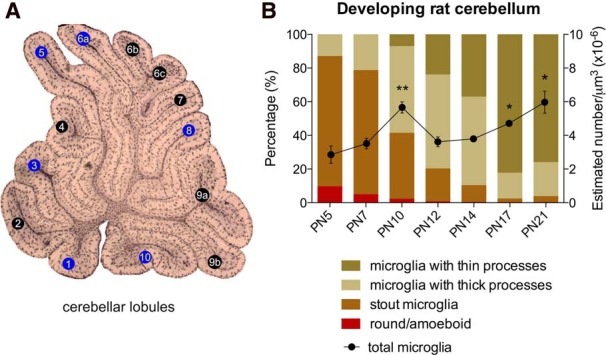
**Postnatal microglia across the developing cerebellum. *A***, A sagittal view of the vermis showing the six lobules used to count microglia (blue background). ***B***, The density of total microglia significantly increased by 1.58-fold in the third postnatal week compared with the first week (*p < 0.05, **p ≤ 0.01 compared with P5; data are expressed as mean ± SEM; *n* = 4, 2 males + 2 females for each group). Colored bars depict the proportion of microglia according to morphology at different time points during postnatal development: round/amoeboid microglia are present but infrequent during the first 10 postnatal days while stout microglia are strongly predominant during the first postnatal week, and microglia with both thick and thin processes are more abundant during the second and third week, respectively.

A design-based unbiased stereological method was performed to quantify microglia, phagocytic cups, and pyknotic bodies across the midvermis. We used StereoInvestigator 10 software (Microbrightfield) interfaced with a Nikon Eclipse 80i microscope and an MBF Bioscience 01-MBF-2000R-F-CLR-12 Digital Camera (Color 12 BIT). Six counting regions (cerebellar lobules 1, 3, 5, 6, 8, and 10; Fig. 1*A*) from every cerebellar section were used for analysis, which are representative of the anterior, posterior, dorsal, and ventral regions of the vermis. A total of four cerebellar sections per animal were used with a physical distance of 225 µm between them. Considering the small size of microglial cells, it is unlikely that any cell was counted twice during the stereological analysis. The optical fractionator probe method was used to estimate cell, phagocytic cup, and pyknotic body densities using a 100 × 100 µm counting frame sampling every 200 µm. We set an optical dissector height of 15 µm with a 2 µm guard zone (top and bottom) to account any change in section thickness during the staining procedure. Both Iba1^+^ cells and phagocytic cups were counted at 20× magnification, and pyknotic bodies counts and the diameter of phagocytic cups at 40× magnification. All quantifications were carried out under blinded experimental conditions. The overall estimated volume of each counting region sampled in the vermis was used to normalize estimated counts to obtain an estimation of the average density of objects of interest (e.g., microglia, phagocytic cups, pyknotic bodies), which was expressed as an estimated number/µm³ (relative density measurement). Although perivascular macrophages are also positive for Iba1, they represent ∼4% of the Iba1^+^ population in the brain (Williamson et al., 2011), suggesting a negligible impact of this factor on the data analysis.

### Developmental profile of microglia and phagocytic cups

Microglia counts were performed on P5, P7, P10, P12, P14, P17, and P21 cerebellums from intact male and female rat pups (*n* = 4, 2 males + 2 females for each group). Microglia were morphologically characterized based on Lenz et al. (2013) with modifications into four categories; (1) round/amoeboid microglia, (2) stout microglia, (3) microglia with thick processes (short or long), and (4) microglia with thin processes (short or long), as also described by others (Wu et al., 1992; Gómez-Gonzalez and Escobar, 2010; Schwarz et al., 2012). For descriptive purposes we identified microglia with thick and thin processes as follows: microglia with thick processes are large cells with an amorphous cell body and with at least two ramified short, long, or both thick processes. Microglia with thin processes are large or small cells with a round and small cell body, with at least four ramified short, long, or both thin processes (more ramified than microglia with thick processes). The density of total microglia, i.e., regardless of morphology, was obtained by summing all four microglial morphologies described above. In addition, cup-shaped invaginations of the plasma membrane formed around cellular debris, infectious agents or dead cells, and called “phagocytic cups” (Swanson, 2008) were also counted in the same cerebellar sections stained with Iba1. To have a clear identification of phagocytic cups, only those located at the tip of microglia processes with a round morphology were counted (Fig. 3*B*,*C*). All counts were performed solely in the cerebellar cortex; the white matter was not included. Cellular layers (granular versus molecular) were not distinguished in this experiment, as they are not fully formed at the younger ages.

### Quantification of microglia and phagocytic cups in the GL and ML

In a separate cohort of animals, microglia and phagocytic cup counts were performed in the granular layer (GL) and the molecular layer (ML) of the cerebellar cortex on P12, P14, P17, and P21 male and female rat pups (*n* = 6, 3 males + 3 females for each group). At these ages both the GL and ML are well developed. Microglia were categorized based on their morphological features as described above and the density of total microglia was also obtained.

### Quantification of phagocytic cups during the third postnatal week of development

In a third animal cohort aged P15, P16, P17, P18, and P19 (*n* = 3 males + 3 females for each group) the phagocytic cups were counted in both the GL and ML. The morphology of microglia associated with the phagocytic cups was also quantified.

### Quantification of pyknotic bodies

Pyknotic bodies were identified using the cresyl violet staining method on *P5, ^P7, *P14, *P17, and *P21 (*n = 6, 3 males + 3 females; ^*n* = 4, 2 males + 2 females). Pyknotic cell quantification was performed using sections from the cerebellar tissues used in the “developmental profile of microglia and phagocytic cups” experiment and followed the same stereological parameters as described above. Likewise, stereological counting was performed in both the GL and ML of the cerebellar cortex.

### Phagocytic cup size

The size of microglia phagocytic cups located in both the GL and ML were measured using the quick circle tool on StereoInvestigator 10 (same microscope and camera specifications described above). The same cerebellar regions (cerebellar lobules) used for stereological counts and four cerebellar slices previously stained for Iba1 from ^P10, *P14, *P17, and *P21 cerebellums (^*n* = 4, 2 males + 2 females; *n = 8, 4 males + 4 females) were used in this assay. This analysis was performed on 16 phagocytic cups per animal, which was then averaged for a single measure per animal.

### Statistical analysis

All data are expressed as mean ± SEM and effect size estimate calculations (η and d) reported in Tables 1 and 2. Developmental profile of microglia and phagocytic cup datasets were analyzed using a one-way ANOVA with age as fixed factor. Datasets from microglia and phagocytic cup counts in the GL and ML, phagocytic cup counts during the third postnatal week of development, Nissl staining and pyknotic bodies counts and phagocytic cup size measurement were analyzed using a two-way ANOVA with age and cerebellar layer as fixed factors. All statistical analysis followed a *post hoc* pairwise comparison using the Holm’s sequential Bonferroni correction to control for familywise error. Sex differences were studied in the microglia and phagocytic cup counts in the GL and ML dataset only at P17 using a Student’s *t* test for each dependent variable. A summary of statistical analysis performed is reported in Table 1 and pairwise comparisons in Table 2. Significance was denoted when *p* ≤ 0.05. All statistical tests were computed in SPSS 22 and graphed using GraphPad Prism 6.

**Table 1.** Summary of statistical analysis*

Statistical table					
	Data structure	Type of test	Test value	Effect size	Power
a	Normally distributed	One-way ANOVA	*F*_(6, 21)_ = 9.559	η = 0.73	0.73
b	Normally distributed	One-way ANOVA	*F*_(6, 21)_ = 8.895	η = 0.72	0.99
c	Normally distributed	One-way ANOVA	*F*_(6, 21)_ = 41.022	η = 0.92	1.00
d	Normally distributed	One-way ANOVA	*F*_(6, 21)_ = 14.074	η = 0.80	1.00
e	Normally distributed	One-way ANOVA	*F*_(6, 21)_ = 93.160	η = 0.96	1.00
f	Normally distributed	One-way ANOVA	*F*_(6, 21)_ = 136.664	η = 0.97	1.00
g	Normally distributed	Two-way ANOVA	*F*_(3, 48)_ = 9.667	η = 0.42	0.99
h	Normally distributed	Two-way ANOVA	*F*_(3, 48)_ = 0.418	—	0.13
i	Normally distributed	Two-way ANOVA	*F*_(3, 48)_ = 2.84	η = 0.18	0.64
j	Normally distributed	Two-way ANOVA	*F*_(3, 48)_ = 0.221	—	0.09
k	Normally distributed	Two-way ANOVA	*F*_(3, 48)_ = 10.517	η = 0.44	0.99
l	Normally distributed	Two-way ANOVA	*F*_(3, 48)_ = 30.770	η = 0.69	1.00
m	Normally distributed	Two-way ANOVA	*F*_(3, 48)_ = 5.521	η = 0.31	0.97
n	Normally distributed	Two-way ANOVA	*F*_(3, 48)_ = 18.271	η = 0.53	1.00
o	Normally distributed	Two-way ANOVA	*F*_(3, 48)_ = 2.860	—	0.38
p	Normally distributed	Two-way ANOVA	*F*_(3, 48)_ = 54.600	η = 0.83	1.00
q	—	—	—	—	—
r	*t* distribution	Student’s *t* test	t_(4)_ = 1.278	—	0.17
s	*t* distribution	Student’s *t* test	*t*_(4)_ = 1.920	—	0.48
t	*t* distribution	Student’s *t* test	*t*_(4)_ = 3.445	d = 3.445	0.73
u	*t* distribution	Student’s *t* test	*t*_(4)_ = 1.000	—	0.12
v	*t* distribution	Student’s *t* test	*t*_(4)_ = 0.496	—	0.1
w	*t* distribution	Student’s *t* test	*t*_(4)_ = 1.683	—	0.26
x	*t* distribution	Student’s *t* test	*t*_(4)_ = 1.858	—	0.30
y	*t* distribution	Student’s *t* test	*t*_(4)_ = 1.044	—	0.13
z	*t* distribution	Student’s *t* test	*t*_(4)_ = 1.726	—	0.27
aa	*t* distribution	Student’s *t* test	*t*_(4)_ = 0.892	—	0.11
bb	*t* distribution	Student’s *t* test	*t*_(4)_ = 1.323	—	0.18

**Table 2.** Summary of pair comparison tests*

**Developmental profile of microglia and phagocytic cups**													
					P7		P10		P12		P14	P17	P21
a	Total microglia		compared with P5		*t*_(6)_ = 4.534		*t*_(6)_ = 4.534 *d* = 3.70		*t*_(6)_ = 1.277		*t*_(6)_ = 1.782	*t*_(6)_ = 4.534 *d* = 3.70	*t*_(6)_ = 4.534 *d* = 3.70
b	Amoeboid microglia		compared with P5		*t*_(6)_ = 1.330		*t*_(6)_ = 2.621 *d* = 2.14		*t*_(6)_ = 3.845 *d* = 3.13		*t*_(6)_ = 4.045 *d* = 3.30	*t*_(6)_ = 4.043 *d* = 3.30	*t*_(6)_ = 4.044 *d* = 3.30
c	Stout microglia		compared with P5		*t*_(6)_ = 0.395		*t*_(6)_ = 1.886		*t*_(6)_ = 6.416 *d* = 5.23		*t*_(6)_ = 7.634 *d* = 6.23	*t*_(6)_ = 8.654 *d* = 7.06	*t*_(6)_ = 8.373 *d* = 6.83
d	Microglia with thick processes		compared with P5		*t*_(6)_ = 2.659 *d* = 2.17		*t*_(6)_ = 7.858 *d* = 6.41		*t*_(6)_ = 8.657 *d* = 7.06		*t*_(6)_ = 5.344 *d* = 4.36	*t*_(6)_ = 1.926	*t*_(6)_ = 1.574
e	Microglia with thin processes		compared with P5		*t*_(6)_ = 1.000		*t*_(6)_ = 5.078 *d* = 4.14		*t*_(6)_ = 3.352 *d* = 2.73		*t*_(6)_ = 4.653 *d* = 3.79	*t*_(6)_ = 25.466 *d* = 20.79	*t*_(6)_ = 31.079 *d* = 25.37
f	Phagocytic cup		compared with P17		P5 *t*_(6)_ = 29.336 *d* = 23.95		P7 *t*_(6)_ = 26.068 *d* = 21.28		P10 *t*_(6)_ = 24.687 *d* = 20.15		P12 *t*_(6)_ = 20.255 *d* = 16.53	P14 *t*_(6)_ = 11.571 *d* = 9.44	P21 *t*_(6)_ = 15.762 *d* = 12.86
**Microglia and phagocytic cups in the ML and GL during postnatal development**													
g	Total microglia		compared with ML		GL *t*_(10)_ = 3.356 *d* = 2.12		GL *t*_(10)_ = 2.640 *d* = 1.66		GL *t*_(10)_ = 3.160 *d* = 1.99		GL *t*_(10)_ = 7.438 *d* = 4.70		
h	Amoeboid microglia		compared with ML		GL *t*_(10)_ = 1.226		GL *t*_(10)_ = 0.049		GL *t*_(10)_ = 1.000		GL *t*_(10)_ = 0.139		
i	Stou*t*microglia		compared with ML		GL *t*_(10)_ = 5.209 *d* = 3.29		GL *t*_(10)_ = 1.192		GL *t*_(10)_ = 3.354 *d* = 2.12		GL *t*_(10)_ = 8.846 *d* = 5.59		
j	Microglia with thick processes		compared with ML		GL *t*_(10)_ = 1.703		GL *t*_(10)_ = 1.326		GL *t*_(10)_ = 0.601		GL *t*_(10)_ = 1.003		
k	Microglia with thin processes		compared with ML		GL *t*_(10)_ = 0.604		GL *t*_(10)_ = 0.116		GL *t*_(10)_ = 2.191 *d* = 1.38		GL *t*_(10)_ = 6.186 *d* = 3.91		
l	Phagocytic cup		compared with ML		GL *t*_(10)_ = 4.013 *d* = 2.53		GL *t*_(10)_ = 2.400 *d* = 1.51		GL *t*_(10)_ = 6.814 *d* = 4.30		GL *t*_(10)_ = 2.279 *d* = 1.44		
**Microglial phagocytic activity during the third postnatal week of development**													
					P15		P16		P18		P19		
m	Phagocytic cup		compared with GL P17		GL *t*_(10)_ = 3.978 *d* = 2.51		GL *t*_(10)_ = 2.198 *d* = 1.39		GL *t*_(10)_ = 0.568		GL *t*_(10)_ = 0.518		
m	Phagocytic cup		compared with ML P17		ML *t*_(10)_ = 1.499		ML *t*_(10)_ = 0.029		ML *t*_(10)_ = 1.300		ML *t*_(10)_ = 2.313 *d* = 1.46		
					P15		P16		P17		P18	P19	
m	Phagocytic cup		compared with the ML		GL *t*_(10)_ = 0.757		GL *t*_(10)_ = 3.052 *d* = 1.93		GL *t*_(10)_ = 4.091 *d* = 2.58		GL *t*_(10)_ = 7.765 *d* = 4.91	GL *t*_(10)_ = 3.348 *d* = 2.11	
**Phagocytic cup size during the third postnatal week of development**													
					P10		P14		P21				
n	Phagocytic cup		compared with P17		*t*_(10)_ = 2.037 *d* = 1.28		*t*_(14)_ = 5.262 *d* = 2.81		*t*_(14)_ = 3.547 *d* = 1.73				
**Pyknotic bodies in the GL than the ML during postnatal development**													
					P5		P14		P17		P21		
p	Pyknotic bodies		compared with GL P7		GL *t*_(8)_ = 0.328		GL *t*_(8)_ = 34.179 *d* = 24.16		GL *t*_(8)_ = 25.394 *d* = 17.95		GL *t*_(8)_ = 39.642 *d* = 28.03		
p	Pyknotic bodies		compared with ML P7		ML *t*_(8)_ = 1.401		ML *t*_(8)_ = 0.416		ML *t*_(8)_ = 0.729		ML *t*_(8)_ = 0.939		
					P5		P7		P14		P17	P21	
p	Pyknotic bodies		compared with ML		GL *t*_(10)_ = 7.163 *d* = 4.53		GL *t*_(6)_ = 19.461 *d* = 15.88		GL *t*_(10)_ = 1.628		GL *t*_(10)_ = 0.100	GL *t*_(10)_ = 1.392	
**Sex differences in microglia morphology on P17 cerebellum**													
						GL		ML					
q/u		Amoeboid microglia		compared with females		males *t*_(4)_ = 0.000		males *t*_(4)_ = 1.000					
r/v		Stout microglia		compared with females		males *t*_(4)_ = 1.278		males *t*_(4)_ = 0.496					
s/w		Microglia with thick processes		compared with females		males *t*_(4)_ = 1.920		males *t*_(4)_ = 1.683					
t/x		Microglia with thin processes		compared with females		males *t*_(4)_ = 3.445 d = 3.445		males *t*_(4)_ = 1.858					
y/z		Phagocytic cup		compared with females		males *t*_(4)_ = 1.044		males *t*_(4)_ = 1.726					
aa/bb		Total microglia		compared with females		males *t*_(4)_ = 0.892		males *t*_(4)_ = 1.323					

## Results

### Microglia increase during the first 3 postnatal weeks of development in an age-specific manner

The density of total microglia significantly increased during postnatal development (*p* < 0.000; Fig. 1*B*; Table 1a. A significant increase in total microglia, compared with P5, was found at P10 (*p* = 0.004), P17 (*p* = 0.012), and P21 (*p* = 0.010), but not at P7 (*p* = 0.322), P12 (*p* = 0.249), or P14 (*p* = 0.125). In addition, the proportion of microglia found in each morphological category changed as the cerebellum developed postnatally. Whereas the proportion of stout microglia were more predominant between P5–P7, the proportion of microglia with both thick and thin processes were more abundant between P10–P14 and P17–P21, respectively (Fig. 1*B*).

### Amoeboid and stout microglia decrease, whereas microglia with both thick and thin processes increase as the cerebellum matures

**Figure 2.**
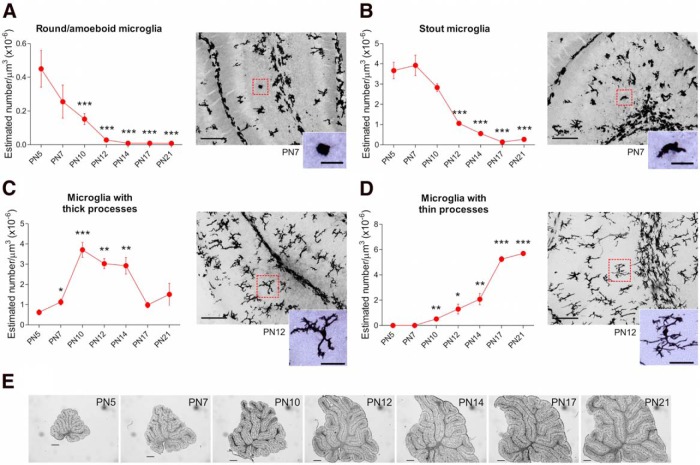
**Morphological profile of microglia in the postnatal developing cerebellum. *A***, The frequency of round/amoeboid microglia significantly decreased after the first postnatal week (***B***), as well as stout microglia. ***C***, Conversely, the density of microglia with thick processes increased only during the second postnatal week followed by a decrease in the third postnatal week. ***D***, The density of microglia with thin processes gradually increased after the first postnatal week doubling their density by the third postnatal week. ***E***, Sagittal views of the midvermis, labeled with Iba1, across the first 3 postnatal weeks. All data are expressed as mean ± SEM (*n* = 4, 2 males + 2 females for each group). Significant differences are detonated by *p < 0.05, **p < 0.01, and ***p < 0.000 compared with P5. Insets depict a higher magnification of selected microglia (red squares) in each panel. Scale bars: gray scale images, 100 µm; color images (inset), 25 µm; ***E***, 500 µm (from P5 to P21). Images in ***A***, ***B***, ***C***, and ***D*** depict the morphology of microglia at two different postnatal ages: P7 (***A***, ***B***) and P12 (***C***, ***D***).

We categorized and counted microglia based on their morphological features across the vermis at different time points during postnatal development. The density of amoeboid microglia were the least common and they significantly decreased after the first postnatal week (*p* < 0.000)^b^. Compared with P5, there were significantly fewer amoeboid microglia at later ages from P10 to P21 (P10, *p* = 0.04; P12, *p* = 0.009; P14, *p* = 0.007; P17, *p* = 0.007; P21, *p* = 0.007; except at P7, *p* = 0.232; Fig. 2*A*). Likewise, the density of stout microglia decreased after the first postnatal week (*p* < 0.000)^c^ from P12 to P21 compared with P5 (P12, *p* = 0.001; P14, *p* < 0.000; P17, *p* < 0.000; P21, *p* < 0.000; but not at P7, *p* = 0.706 or P10, *p* = 0.108; Fig. 2*B*). In contrast, the density of microglia with thick processes significantly increased after the first week (*p* < 0.000)^d^ with there being more on P7 (*p* = 0.038), P10 (*p* < 0.000), P12 (*p* < 0.000), and P14 (*p* = 0.002) compared with P5. However, by the third postnatal week (*P17 and ^P21) the density of thick processed microglia dropped back down to immature levels (*p = 0.102 and ^*p* = 0.167, respectively; Fig. 2*C*). By contrast, the density of microglia with thin processes steadily increased as the cerebellum developed (*p* < 0.000)^e^ with a significant difference from P10 until P21 (P10, *p* = 0.002; P12, *p* = 0.015; P14, *p* = 0.003; P17, *p* < 0.000; P21, *p* < 0.000 compared with P5; Fig. 2*D*).

### The density of microglia phagocytic cups peaks during the third postnatal week of cerebellar development

**Figure 3.**
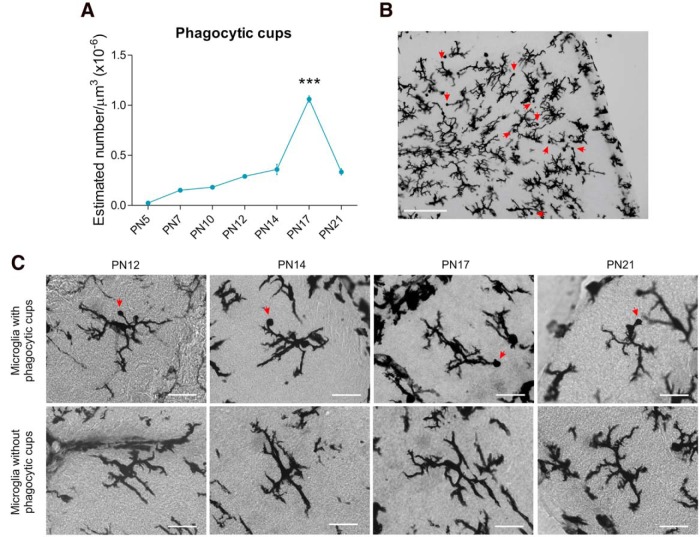
**Microglial phagocytosis in the postnatal developing cerebellum. *A***, The highest density of phagocytic cups was observed during the third postnatal week at P17 (***p < 0.000 compared with P5, P7, P10, P12, P14, and P21; *n* = 4, 2 males + 2 females for each group). Data are expressed as mean ± SEM. ***B***, Phagocytic cups exhibited by microglia (red arrows) in the developing cerebellum at P17. Scale bar, 100 µm. ***C***, Microglia with phagocytic cups (top) or microglia without phagocytic cups (bottom row) at different time points during postnatal development. Scale bars, 25 µm.

The frequency of phagocytic cups changed across development (*p* < 0.000)^f^, with the highest density observed on P17 compared with each time point in this experiment (P5, *p* < 0.000; P7, *p* < 0.000; P10, *p* < 0.000; P12, *p* < 0.000; P14, *p* < 0.000; P21, *p* < 0.000; Fig. 3*A*).

### There are more microglia in the GL than the ML in the cerebellar cortex

**Figure 4.**
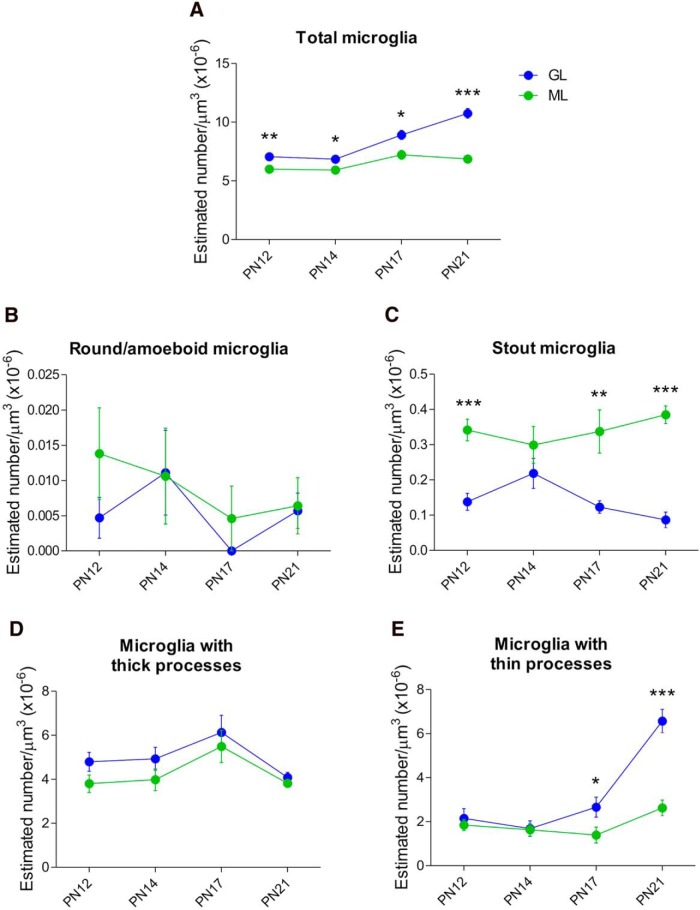
**Microglia location in the cerebellar cortex based on morphological classification. *A***, Total microglia were significantly higher in the GL than the ML during the second and third postnatal week in the cerebellum (*p < 0.05, **p < 0.01, ***p < 0.000). ***B***, The density of round/amoeboid microglia was very low and did not differ between the ML and the GL from P12 to P21. ***C***, The density of stout microglia was significantly higher in the ML than the GL at all days examined except P14 (**p < 0.01, ***p < 0.000). ***D***, Microglia with thick processes were the most abundant but did not differ between the ML and the GL. ***E***, There were significantly more microglia with thin processes in the GL than the ML at P17 and P21 but not at younger ages examined (*p < 0.05, ***p < 0.000). All data are expressed as mean ± SEM (*n* = 6, 3 males + 3 females for each group).

To test whether the density of total microglia and/or their morphology differs based on anatomical location in the cerebellar cortex, we counted microglia separately in both the GL and ML at different time points during postnatal development. A significant interaction for age X cerebellar layer was found for total microglia (*p* < 0.000)^g^. The GL layer had a higher density of total microglia compared with the ML at P12 (*p* = 0.007), P14 (*p* = 0.025), P17 (*p* = 0.010), and P21 (*p* < 0.000; Fig. 4*A*). When we looked at the microglial morphology, there was a significant interaction of age X cerebellar layer for stout microglia (*p* = 0.05)^i^. The ML exhibited a higher density of stout microglia than GL at P12 (*p* < 0.000), P17 (*p* = 0.007) and P21 (*p* < 0.000), but not at P14 (*p* = 0.261) (Fig. 4*C*). Likewise, a significant interaction for age X cerebellar layer was detected for microglia with thin processes (*p* < 0.000)^k^. We found the GL to have higher density of microglia with thin processes than the ML later in development (P17; *p* = 0.05, P21; *p* < 0.000), but not earlier (P12; *p* = 0.56, P14; *p* = 0.91; Fig. 4*E*). No significant interactions for age X cerebellar layer were found for round/amoeboid microglia (*p* = 0.74; Fig. 4*B*)^h^ or for microglia with thick processes (*p* = 0.88; Fig. 4*D*)^j^.

### Phagocytic cups are more frequent in the GL than the ML of the vermis during the third postnatal week

**Figure 5.**
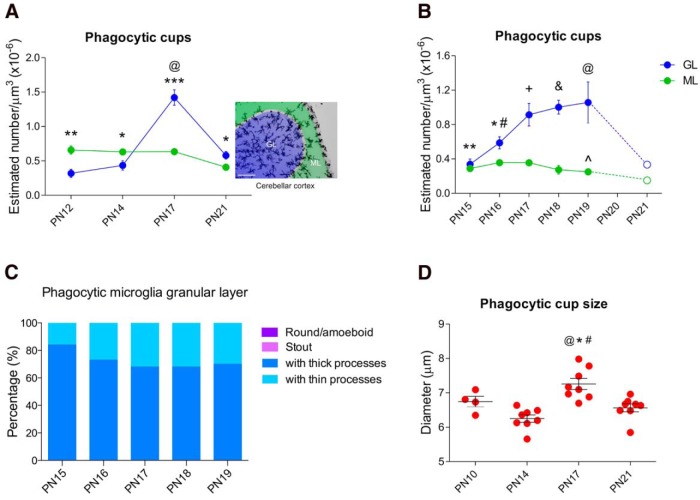
**Frequency of phagocytosis by microglia changes by location in the cerebellar cortex across development. *A***, The density of phagocytic cups was higher in the ML than the GL at P12 (**p < 0.01), and P14 (*p < 0.05), but switched at P17 (***p < 0.000) and P21 (*p < 0.05), so that the GL exhibited more phagocytic cups than the ML. The highest density of phagocytic cups was found in the GL at P17 compared with P12, P14, and P21 (^@^*p* < 0.000). Scale bar, 100 µm. ***B***, Proportion of microglia that exhibited phagocytic cups in the GL at P17: 67% of all phagocytic microglia had thick processes and 33% had thin processes. No round/amoeboid or stout microglia showed phagocytic cups. ***C***, A difference in the density of phagocytic cups was found at younger ages (P15, **p = 0.003; P16, *p = 0.05) compared with P17, but no significant differences were found at older ages (P18, *p* = 0.583; P19, *p* = 0.615). In contrast, in the ML, the density of phagocytic cups was lower only at P19 (^*p* = 0.043) compared with P17. Additionally, a difference in the density of phagocytic cups between the GL and ML was found from P16 to P19 (P16, ^#^*p* < 0.000; P17; ^+^*p* < 0.000; P18, ^&^*p* < 0.000; P19, ^@^*p* < 0.000) but not at P15 (*p* = 0.467) (*n* = 6, 3 males + 3 females for each group for ***A***, ***B***, and ***C***). In this experiment the density of phagocytic cups was not counted in animals at P21 but the dashed lines depict the pattern previously observed at the end of the third postnatal week in both the GL and ML (Fig. 5*A*). ***D***, The diameter of microglial phagocytic cups was bigger on P17 compared with P10 (^@^*p* = 0.06; see effect size estimation in Table 2), P14 (*p < 0.000) and P21 (^#^*p* = 0.003). All data are expressed as mean ± SEM (^*n* = 4, 2 males + 2 females; *n = 8, 4 males + 4 females: ^P10, *P14, *P17, and *P21).

A significant interaction for age X cerebellar layer for phagocytic cups was also found (*p* < 0.000)^l^. *Post hoc* pairwise comparison revealed a higher density of phagocytic cups in the ML than the GL at younger ages (P12, *p* = 0.002; P14, *p* = 0.037). However, this pattern reversed at slightly older ages with the GL exhibiting more phagocytic cups than the ML at P17 (*p* < 0.000) and P21 (*p* = 0.046) (Fig. 5*A*).

Because phagocytic cups in the developing cerebellum were highest at P17 in the GL (Figs. 3*A*, 5*A*), we sought to determine whether this peak was exclusive to that age or more broadly present. Therefore, we counted phagocytic cups on 2 consecutive days before and after P17 in both the ML and the GL of the cerebellar cortex. There was a significant interaction for age X cerebellar layer in phagocytic cups (*p* = 0.001)^m^. *Post hoc* pairwise comparisons revealed the density of phagocytic cups in the GL was lower at P15 (*p* = 0.003) and P16 (*p* = 0.05) compared with P17 (Fig. 5*B*). No significant differences were found for phagocytic cup density at P18 (*p* = 0.583) or P19 (*p* = 0.615) compared with P17 (Fig. 5*B*), indicating a plateau from P17 to P19. In contrast, in the ML the density of phagocytic cups significantly decreased at P19 compared with P17 (*p* = 0.043; Fig. 5*B*). Additionally, we replicated the significant difference between the GL and ML in terms of phagocytic cup density. The GL had a greater density of phagocytic cups than the ML from P16 to P19 (P16, *p* = 0.012; P17, *p* = 0.002; P18, *p* < 0.000; P19, *p* = 0.007; but not at P15, *p* = 0.467; Fig. 5*B*).

Additionally, we found the phagocytic cups located in the GL associated exclusively with ramified microglia, with thick and thin processes, from P15 to P19 (Fig. 5*C*). During this timeframe, the proportion of phagocytic microglia with thick processes (≥68%) was higher than the proportion of phagocytic microglia with thin processes (≥16%). By P17, the proportion of microglia with thick processes decreased ∼16% keeping that proportion until P19. In contrast, the proportion of microglia with thin processes increased ∼17% between P15 and P17 maintaining a similar proportion of cells until P19 (Fig. 5*C*).

### Phagocytic cups are largest during the third postnatal week in both the GL and ML

We quantified the size of individual phagocytic cups and a 2 × 4 ANOVA analysis detected a significant main effect of age (*p* < 0.000)^n^. Pairwise comparisons revealed P17 cerebellums have larger phagocytic cups than those found at P10 (*p* = 0.06), P14 (*p* < 0.000), and P21 (*p* = 0.003; Fig. 5*D*). There was no impact of cerebellar layer on cup size (*p* = 0.09)^o^.

### Pyknotic bodies are more prevalent in the GL than the ML only during the first postnatal week

**Figure 6.**
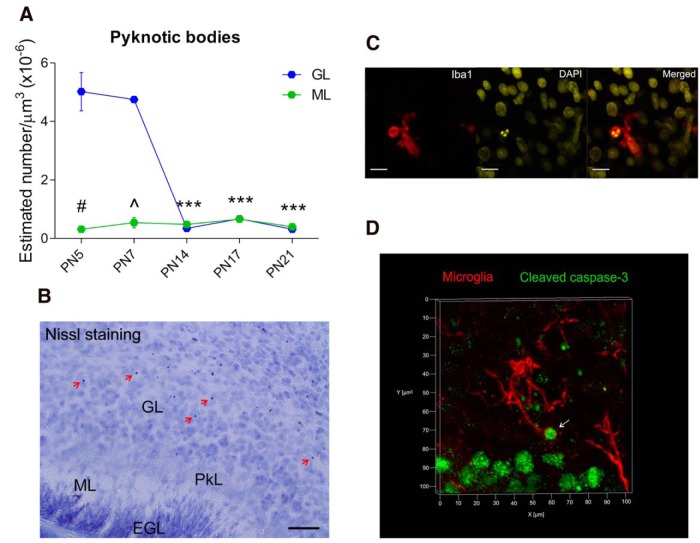
**Identification of pyknotic bodies by Nissl staining in the postnatal developing cerebellum. *A***, The density of pyknotic bodies (red arrows) decreased only in the GL after the first postnatal week at P14, P17 and P21 (***p < 0.000), but not at P7 (*p* = 0.302), compared with P5. No changes in the density of pyknotic bodies were detected in the ML across the developmental time points analyzed when compared with P7 (P5, *p* = 0.199; P14, *p* = 0.688; P17, *p* = 0.487; P21, *p* = 0.375). The GL exhibited more pyknotic bodies than the ML only during the first postnatal week at P5 (^#^*p* < 0.000) and P7 (^*p* < 0.000). Scale bar, 25 µm. Data are expressed as mean ± SEM (*n = 6, 3 males + 3 females; ^*n* = 4, 2 males + 2 females: *P5, ^P7, *P14, *P17, and *P21). ***B***, P7 cerebellar sagittal section stained with cresyl violet showing pyknotic bodies pointed out by red arrows. Pk, Purkinje layer; EGL, external granular layer. ***C***, Confocal colocalization of a pyknotic body (fragmented nucleus in yellow) and a phagocytic cup (red) in the cerebellar cortex at P17. Scale bars, 15 µm. ***D***, 3D confocal image depicting a colocalization of a microglial phagocytic cup (red) and a cleaved caspase-3-positive cell (green) at the tip of a microglia process (white arrow).

We quantified the density of pyknotic bodies in the cerebellar cortex (Fig. 6*B*) at different postnatal time points to establish a pattern of cell death and to see whether it correlated with the pattern of increased phagocytosis at P17. A significant interaction between age X cerebellar layer for pyknotic bodies was detected (*p* < 0.000)^p^. Pyknotic bodies density decreased in the GL at P14 (*p* < 0.000), P17 (*p* < 0.000) and P21 (*p* < 0.000) compared with P7, but not at P5 (*p* = 0.751; Fig. 6*A*). No changes in the density of pyknotic bodies were detected in the ML in any of the developmental time points analyzed compared with P7 (P5, *p* = 0.199; P14, *p* = 0.688; P17, *p* = 0.487; P21, *p* = 0.375; Fig. 6). Moreover, there were more pyknotic bodies in the GL than the ML at P5 (*p* < 0.000) and P7 (*p* < 0.000), but not at later ages (P14, *p* = 0.134; P17, *p* = 0.922; P21, *p* = 0.194; Fig. 6*A*). These data indicate there is not a clear relationship between the appearance of both phagocytic cups and pyknotic bodies in the developing cerebellum.

We also found colocalization of pyknotic cell bodies within some phagocytic cups in the cerebellar cortex at P17 (Fig. 6*C*). As expected, the cell death marker cleaved caspase-3 also colocalized with some phagocytic cups at P17 (Fig. 6*D*). However, we also detected cleaved caspase-3 broadly in the cerebellar cortex and white matter, with an intense expression in the Purkinje cell layer. This did not appear related to cell death (see Discussion).

### Males have more microglia with thin processes than females in the GL at P17

**Figure 7.**
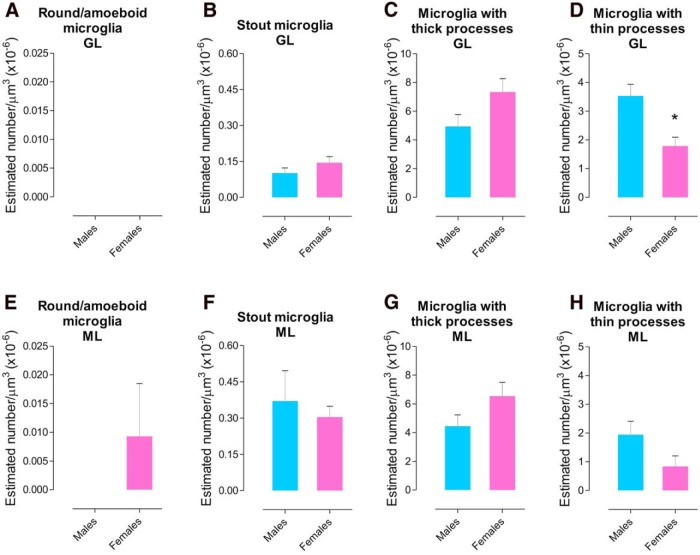
**Microglial sex differences in the developing cerebellum. *A***–***D***, Estimated density of microglia based on morphology in the GL at P17. Males had more microglia with thin processes than females (*p = 0.026). No significant differences were found for sex in round/amoeboid microglia, stout microglia (*p* = 0.270) or microglia with thick processes (*p* = 0.127). E–H, Estimated density of microglia based on morphology in the ML at P17. The statistical analysis indicated no sex differences in round/amoeboid (*p* = 0.375), stout (*p* = 0.646), microglia with thick processes (*p* = 0.168), or microglia with thin processes (*p* = 0.137). Data are expressed as mean ± SEM (*n* = 6, 3 males + 3 females for each group).

We looked at sex differences in the cerebellum in terms of microglia density at P17 in both the GL and ML to determine whether at this unique time point the cerebellum develops differently according to sex. We found that males have a higher density of microglia with thin processes than females in the GL (*p* = 0.026^t^; Fig. 7*D*). No significant differences were found in any other of the morphological categorization of microglia in the GL (round/amoeboid; *t*_(4)_ = 0.000^q^; stout, *p* = 0.270^r^; with thick processes, *p* = 0.127^s^; Fig. 7*A*–*C*) or the ML (round/amoeboid, *p* = 0.375^u^; stout, *p* = 0.646^v^; with thick processes, *p* = 0.168^w^; with thin processes, *p* = 0.137^x^; Fig. 7*E*–*H*). Also, there were no significant differences between males and females for phagocytic cups in the GL (*p* = 0.356)^y^ or ML (*p* = 0.159)^z^. The same results were found for total microglia (GL, *p* = 0.423^aa^; ML, *p* = 0.256^bb^).

## Discussion

The rat cerebellum reaches maturation during the first 3 postnatal weeks and undergoes remarkable anatomical changes during this time (Heinsen, 1977; Altman, 1982; Goldowitz and Hamre, 1998; Sotelo, 2004; Sillitoe and Joyner, 2007; Butts et al., 2014). We found microglia also dramatically change their morphological profile from round to ramified in the cerebellar cortex during this dynamic period of development (Fig. 1*B*). This developmental profile of microglia in the cerebellum is consistent with previous findings in other regions of the CNS, i.e., going from round to ramified morphology (Wu et al., 1992; Schwarz et al., 2012). However, morphological differences in microglia varied by subregion (ML vs GL), suggesting an important role for local factors in regulating differentiation. A decline in round/amoeboid microglia after the first postnatal week in the entire mouse cerebellum has also been reported, although with a different developmental profile (Ashwell, 1990). The frequency of stout microglia also declined along a similar time course to the amoeboid, whereas microglia with both thick and thin processes increased as the cerebellum matured. These data indicate a continuous process of microglial maturation during the first 3 postnatal weeks that might be related to a specific function as the cerebellum develops including regulation of cell death and synaptic pruning.

Microglia remove apoptotic cellular debris through phagocytosis (Parnaik et al., 2000; Neumann et al., 2009; Sierra et al., 2010, 2013). Phagocytosis by ramified microglia (with thick or thin processes) requires the formation of round structures of actin called phagocytic cups (Swanson, 2008). The appearance of a marked increase in phagocytic cups during the third postnatal week, particularly at P17, suggests this is a critical period for microglial phagocytosis in the developing cerebellum. Moreover, the content of some phagocytic cups indicated pyknotic bodies, suggesting the engulfment of apoptotic cells, as has been observed in other regions of the CNS (Sierra et al., 2010). Previous evidence of microglial phagocytosis in the developing cerebellum focused on round or amoeboid microglia (Ashwell, 1990). Here we show that ramified microglia are the dominant committers of phagocytosis during the second and third postnatal week of development in the cerebellum. However, how phagocytic microglia contribute to the establishment of the cerebellar circuit remains poorly understood.

The cerebellar cortex is organized into three anatomical layers that contain different types of neurons with specific developmental timeframes and populations (Altman, 1982; Sotelo, 2004). The GL consists of a larger variety and population of cells compared with the ML and the Purkinje layer that only contains two different interneurons and one type of neuron, respectively (Burgoyne and Cambray-Deakin, 1988; Voogd and Glickstein, 1998; Apps and Garwicz, 2005). We observed differences between the GL and ML in terms of microglial population. The overall number of microglia are consistently higher in the GL than the ML from P12 to P21, but interestingly, this pattern changes when the morphology of microglia is taken into account. Although more stout microglia are present in the ML than the GL during the second and third postnatal week, there are more microglia with thin processes in the GL than the ML, but only during the third postnatal week. Stout microglia are still differentiating and changing into the ramified form as part of their maturation likely in their final location in the cerebellar cortex. On the other hand, microglia with thin processes have already reached their final location in the cerebellar cortex increasing in cell density as the cerebellum matures. Thus, our findings indicate that microglia are not uniformly distributed in the cerebellar cortex of the developing cerebellum, a finding consistent with a previous report in the young (>25 d) and adult mouse cerebellum (>90 d; Vela et al., 1995).

A surprising observation was the high degree of regional and temporal specificity of the phagocytic activity of microglia, an important function for the normal developing brain as well as in the adult brain under infectious or damage situations (Neumann et al., 2009; Sierra et al., 2013). Phagocytosis by microglia was higher in the ML during the second postnatal week but a few days later the GL had dramatically more phagocytic cups until the end of the third week. This switch may be driven by a combination of processes occurring in the GL required for its maturation, such as cell proliferation and formation of synapses (Altman, 1972; Burgoyne and Cambray-Deakin, 1988; Carletti and Rossi, 2008), but also cell death.

We found a peak of phagocytic cups density at P17, which was localized to the GL and persisted for a 3 d window in the third postnatal week of development. During this window the proportion of microglia with thick processes decreased ∼16%, whereas the proportion of microglia with thin processes increased ∼17%, suggesting a final maturation phase. Although this change is related to maturation of the cerebellum, whether it is critical for the establishment of the GL remains unknown. We found no evidence to suggest the increased phagocytosis at P17 was solely for the removal of dead cells since the pattern of pyknotic bodies, a measure of cell death, bore no resemblance to the pattern of microglial phagocytosis. Similar results in the mouse developing cerebellum are in accordance with ours (Wood et al., 1993; Lossi et al., 2002). Nonetheless, the colocalization of pyknotic bodies and cleaved caspase-3 with some phagocytic cups at P17 in the cerebellar cortex indicates that microglia are removing apoptotic cells. Interestingly, our observation of cleaved caspase-3 expression broadly in the cerebellum suggests it may be participating in nonapoptotic processes, such as cell differentiation, cell proliferation, neurite pruning, and synaptic plasticity as described by others (Oomman et al., 2004; D'Amelio et al., 2012; Hyman and Yuan, 2012; Shalini et al., 2015). The removal of apoptotic cells by ramified microglia is supported by the size of the phagocytic cups, which correlates with the size of granule neurons (Burgoyne and Cambray-Deakin, 1988), the only neurons proliferating during the second and third postnatal weeks in the cerebellum (Carletti and Rossi, 2008). Nevertheless, synaptic and axonal debris could also undergo removal by microglia as part of the synaptic changes and maturation of the climbing and mossy fibers at this age (Goldowitz and Hamre, 1998; Hashimoto and Kano, 2005; McKay and Turner, 2005).

Microglia regulate synapses and axons during development in other regions of the CNS during the second and third postnatal weeks (Berbel and Innocenti, 1988; Tremblay et al., 2010; Paolicelli et al., 2011; Schafer et al., 2012). This implicates microglia as being directly involved in the remodeling of neural synaptic circuits. By the second postnatal week rat pups generally open their eyes (Reiter et al., 1975) and also begin to respond to auditory signals (Friauf, 1992). By the third postnatal week play behavior appears (Meaney and Stewart, 1981; Panksepp, 1981; Auger and Olesen, 2009).Thus, the cerebellum processes motor and sensorial stimulation that may regulate synaptic connections and therefore, the phagocytic activity of microglia.

We measured the size of phagocytic cups and found them to differ according to age, but not to location in the cerebellar cortex. The size of the cups was largest on P17, when phagocytosis peaks in the GL, but not in the ML. This finding suggests that phagocytic microglia are engulfing either larger or greater amounts of cellular debris at P17. The difference between the largest and the smallest phagocytic cup was equal to 1 µm, which might indicate a precise and efficient process of phagocytic cup formation. However, whether this difference carries a significant biological function is not clear.

Evidence in this study supports the conclusion that P17 is an important time point for microglia function in the development of the cerebellum. This function depends on the location of microglia in the cerebellar cortex, but we also found sex to influence microglia in the cerebellum as males showed more microglia with thin processes than females in the GL, but not in the ML, at P17. Microglia with thin processes presumably have reached their final location in the cerebellar cortex; therefore, they are matured and surveying their local environment suggesting a different pattern in microglial maturation in the GL according to sex that might influence the assembly of the cerebellar circuit. However, there was no sex difference in frequency of phagocytic cups in the cerebellar layers at this age, indicating that phagocytosis by microglia is similar between males and females in the developing cerebellum. Sex differences have been reported in both the adult human and rat cerebellums in terms of anatomy and function (Dean and McCarthy, 2008). Here we show a sex difference at the cellular level in a structure vulnerable to damage during development. The cerebellum is commonly altered in developmental disorders, such as autism, a disorder with gender bias in its prevalence (Bauman and Kemper, 2005; Amaral et al., 2008; Perez-Pouchoulen et al., 2012; Werling and Geschwind, 2013). Therefore, these results might give insights to address other ways to explore the developing cerebellum under normal and abnormal conditions.

Altogether, this work contributes to the understanding of the role of microglia in the rat cerebellum during normal development with a particular focus on the development of the vermis, a vulnerable structure to developmental alterations. To understand what is inadequate in the abnormal developing cerebellum we have to understand first how the cerebellum is formed and the contribution of microglia to this end.

## Synthesis

The decision was a result of the Reviewing Editor Christian Luscher and the peer reviewers coming together and discussing their recommendations until a consensus was reached. A fact-based synthesis statement explaining their decision and outlining what is needed to prepare a revision is listed below. The following reviewers agreed to reveal their identity: Nina Swinnen, Michael Dailey

I would like the authors to address all points raised be the referees by re-analyzing their data and make the appropriate changes in the manuscript.

Reviewer #1:

The manuscript provides information on microglial cell behavior in the postnatal rat cerebellum. An important structure in neurdevelopmental disorders. Hence this information is essential in the further research on the role of microglial cells in cerebellum development and their contriubution to disorders as autism.

Althoug clearly written I have some remarks/questions for the authors.

Mat.&amp;metods:

IHC: Consider changing terminology 'until they sank' to 'until they were saturated'.

What was the rationale for selecting the six counting regions/lobules used in the study?

Please explain based on what measurement/defenition microglual cell processes were considered to have thick vs thin processes

Results/Figures:

Please provide the morphological quantifications or p values for fig 1b

Please give N in the figure legends

To my opinion it will more clear for the readers if the mean +- SEM is provide in the text in stead of the listings as provided now with size estimate calculation. This makes the manuscript sometimes very messy/difficult to read.

Please indicate clearly in the text when you are talking about absolute numbers or percentages/proportions, this is not always clear.

Was there a difference in absolute counts of microglia between the GL and ML? The subtitle suggests this but it is not mentioned in the text.

Were stainings for cell death performed,f.e. cleaved caspase 3? In addition, did the authors try to identify the cells that were pyknotic/contacted by the phagocytic cups? If so this would greatly increase the value of the paper.

Reviewer #2:

The question of the role of microglia in cerebellar development is potentially interesting and important, given recent work indicating active roles for microglia in neural development in other brain regions. The current work uses staining (Iba1), imaging, and quantitative morphometry to assess microglial density and morphology across postnatal cerebellar development in the rat. The authors conclude (p. 1) that their findings "indicate a continuous process of microglial maturation and a non-uniform distribution of microglia in the cerebellar cortex that implicates microglia as an important cellular component of the developing cerebellum."

The manuscript is written in a generally clear and readable prose. The figures are clear and attractive. The statistical analyses seem rigorous, with a few exceptions. The major problem with the work is its limited scope, being highly descriptive with no experimental manipulations to truly test a falsifiable hypothesis or to get at mechanism or function. The authors' conclusions are based on correlational data and are sometimes vague or speculative. For example, the authors state (p.2) that "this study...contributes to the growing appreciation of how microglia function during postnatal development," but the results really do not provide any significant step forward. Based on the data presented, it may be that microglia phagocytosis only represents a clearance activity. Again, the authors state (p. 15) that they show "that ramified microglia are the dominant committers of phagocytosis in the developing cerebellum and this may be a component of the appropriate establishment of the cerebellar circuit." While this function of ramified is evident (as also shown in previous studies), the current study provides no firm basis for establishing the link between microglial phagocytosis and specific aspects of cerebellar circuit development or plasticity.

The authors' hypothesis is that "microglia change their morphological profile and cell number in direct relationship to the maturation of the cerebellum as well as the anatomical location within the cerebellar cortex." But given the current literature, what reasonable alternative hypotheses are there? Potentially, the observations could identify spatial-temporal patterns of activity of microglia phagocytosis of apoptotic cells or axon terminals/synapses, but the results suggest that neuronal cell death does not correlate well with microglial phagocytic activity (surmised from phagocytic cup frequency). This leaves open the significance of the observations. As such, the study does not significantly extend our understanding of the role of microglia in cerebellar (or brain) development.

Other specific issues:

1) The authors typically present a summary of their data before presenting the raw data. It is preferable, at least to this reader, to see the raw data before any summary or statistical analyses. For example, Fig. 1B provides a quantitative summary of the various morphological types of microglia, but examples of each morphological type are not presented until Fig. 2. The same is true for other analyses, including the phagocytic cups. Moreover, the authors should present representative examples of larger fields of view showing the range of morphological types of microglia, not just cropped images of isolated cells.

2) Categorization of microglia is based on image analysis using the StereoInvestigator software. Although another study is referenced, the authors should state in the present manuscript how they defined "thick" and "thin" processes? It seems that this delineation could significantly alter the outcome. Moreover, the authors need to consider and discuss how any image processing may affect the morphometric analysis. For example, how does altering the brightness/contrast of the images affect the categorization of microglia with thick vs. thin processes? Could it be that phagocytic cups are more evident in microglia with "thin processes" and are rather obscured in the "thick processes" of microglia? Could this explain why there is a large spike in phagocytic cups at PN17, a time when microglia with "thin processes" begin to predominate?

3) Related to this point, from the images shown it appears that microglia at PN12 have the thinnest processes (Fig. 3B), yet the quantitation in Fig. 1 indicate that microglia with thin processes predominate later in development.

4) The authors should describe the criteria they use for identifying "phagocytic cups." What is the lower limit in size of the structures they consider "cups"? Given that all of these phagocytic cups are expected to shrink and become completely resorbed during the degradation phase, what is the significance of the "average size" of the cups? As mentioned by the authors, the size of the largest cups suggests engulfment of fairly large structures such as cell bodies, rather than synaptic terminals. Small cups may simply represent shrunken larger cups. It would be helpful to indicate the absolute number of phagocytic cups counted, not just the average density. This could be done by showing a scatter plot of the individual cup sizes in addition to the mean values in Fig. 5D.

5) The authors frequently use the term "number" when they may actually mean "density." E.g., p. 9, line 2, and the second bold heading on p. 10 ("more microglia" could refer to absolute number of microglia in a larger tissue volume or to higher cell density).

6) The authors state (p. 17-18) that "sex is a factor that influences microglia in the cerebellum as males showed more microglia with thin processes than females..." Did the authors assess whether the size or weight of the animals was correlated with microglial density, or consider whether this could account for an apparent sex-link effect?

Reviews of eN-NWR-0036-15

Dear Dr. Luscher,

We are pleased to learn that our manuscript is potentially acceptable for publication in eNeuro. We thank the reviewers and you for thoughtful and constructive critiques which have allowed us to improve the manuscript. Our point-by-point responses to the reviews can be found below and we note any changes in the manuscript in BOLD. We look forward to hearing your response and future publication in eNeuro.

Reviewer #1:

The manuscript provides information on microglial cell behavior in the postnatal rat cerebellum. An important structure in neurodevelopmental disorders. Hence this information is essential in the further research on the role of microglial cells in cerebellum development and their contribution to disorders as autism.

We thank the reviewer for these positive comments.

IHC: Consider changing terminology 'until they sank' to 'until they were saturated'.

We have changed the terminology in the sentence as suggested (page 4): "cryoprotected with 30 % sucrose until they were saturated".

What was the rationale for selecting the six counting regions/lobules used in the study?

We selected those six cerebellar lobules in order to have a representation of the ventral and dorsal parts of the vermis as well as the anterior and posterior ones. This was stated in the manuscript as follows (page 6): "These counting regions are representative of the anterior, posterior, dorsal and ventral regions of the vermis".

Please explain based on what measurement/definition microglial cell processes were considered to have thick vs thin processes

We have stated in the manuscript our criteria to define microglia with thick and thin processes (page 7) as follows: "For descriptive purposes we identified microglia with thick and thin processes as follows: microglia with thick processes are generally large cells with an amorphous cell body, and with generally ramified short or long thick processes. Whilst, microglia with thin processes are large or small cells with a round and small cell body, with ramified short or long thin processes (more ramified than microglia with thick processes)".

Results/Figures: Please provide the morphological quantifications or p values for fig 1b

The color bars in Figure 1B are intended to be descriptive; therefore, no morphological quantifications or p values are provided. However, the same data is now presented as total microglia and the amount of each morphological category in Figure 2 and p values are provided in pages 9-10 and 23-24 of the manuscript.

Please give N in the figure legends

We now include the number of animals used in each figure legend (pages 23-26).

To my opinion it will be more clear for the readers if the mean +- SEM is provide in the text instead of the listings as provided now with size estimate calculation. This makes the manuscript sometimes very messy/difficult to read.

We have extended the information provided in Table 1 and also created an additional Table 2 in order to provide all the statistical information and thereby make the manuscript easier to read. However, we did not include the mean {plus minus} S.E.M. in the results section in order to maintain easy reading as they are presented in the graphs.

Please indicate clearly in the text when you are talking about absolute numbers or percentages/proportions, this is not always clear.

We have clarified our writing in the text of the manuscript as suggested by the reviewer.

Was there a difference in absolute counts of microglia between the GL and ML? The subtitle suggests this but it is not mentioned in the text.

Yes, there is a difference in the density of total microglia between the GL and ML and it has been added in both the Results (page 11) and Discussion sections (page 15) as well as in Figure 4A. The information is stated as follows:

Results: "A significant interaction for age X cerebellar layer was found for total microglia (p &lt; 0.000)g. The GL layer had a higher density of total microglia compared to the ML at PN12 (p = 0.007), PN14 (p = 0.025), PN17 (p = 0.010) and PN21 (p &lt; 0.000) (Figure 4A). When we looked at the microglial morphology...".

Discussion: "The overall microglia are consistently higher in the GL than the ML from PN12 to PN21, but interestingly, this pattern changes when the morphology of microglia is taken into account".

Were stainings for cell death performed, i.e. cleaved caspase 3? In addition, did the authors try to identify the cells that were pyknotic/contacted by the phagocytic cups? If so this would greatly increase the value of the paper.

We agree with the Reviewer and indeed, we performed an immunohistochemistry for the activated caspase-3 (Asp175) in cerebellar sections and it has been included in both Methods, Results and Discussion sections of the manuscript (pages 5, 13 and 16-17) as well as in Figure 6 (6D). We stated such information as follows:

Methods: "In order to co-localize dead cells with microglial phagocytic cups, we followed the fluorescence protocol described above to identify the cellular death marker caspase-3 (1:750, Cell Signaling Technology) and Iba1 (1:1000, Abcam) on PN17 cerebellar sections. Anti-rabbit Alexa FluorÂ® 488 dye (1:500; Invitrogen) and anti-goat Alexa Fluor 594Â® dye (1:500, Invitrogen) were used as secondary antibodies".

Results: We also found a co-localization of some pyknotic bodies with some phagocytic cups in the cerebellar cortex at PN17 (Figure 6C). As expected, the cell death marker caspase-3 also co-localized with some phagocytic cups at PN17 (Figure 6D). It is noteworthy that caspase-3 was located all over the cerebellar cortex and white matter, with an intense expression in the Purkinje cell layer.

Discussion: "Nonetheless, the co-localization of pyknotic bodies and caspase-3 with a phagocytic cup at PN17 in the cerebellar cortex, indicates that microglia are removing apoptotic cells". Interestingly, our observations of caspase-3 all over the immature cerebellum suggest that caspase-3 may be participating in non-apoptotic processes such as cell differentiation or cell proliferation as described by others [Oomman et al., 2004; D'Amelio et al., 2012; Hyman and Yuan, 2012; Shalini et al., 2015]. The removal of apoptotic cells by ramified microglia.

We also tried to determine whether granule neurons were contacted by phagocytic cups at PN17 since they are the most abundant cells in the granular layer, where the highest density of phagocytic cups was found at PN17. Moreover, granule neurons are still migrating during the third postnatal week. Toward this end, we used Pax6 and ZNF-38 as granule neuron markers; however, the high cell density in the granular layer made it very difficult to identify co-localization of a phagocytic cup with a granule neuron marker and so we are exploring other approaches.

Reviewer #2:

The question of the role of microglia in cerebellar development is potentially interesting and important, given recent work indicating active roles for microglia in neural development in other brain regions. The current work uses staining (Iba1), imaging, and quantitative morphometry to assess microglial density and morphology across postnatal cerebellar development in the rat. The authors conclude (p. 1) that their findings "indicate a continuous process of microglial maturation and a non-uniform distribution of microglia in the cerebellar cortex that implicates microglia as an important cellular component of the developing cerebellum."

The manuscript is written in a generally clear and readable prose. The figures are clear and attractive. The statistical analyses seem rigorous, with a few exceptions. The major problem with the work is its limited scope, being highly descriptive with no experimental manipulations to truly test a falsifiable hypothesis or to get at mechanism or function. The authors' conclusions are based on correlational data and are sometimes vague or speculative. For example, the authors state (p.2) that "this study...contributes to the growing appreciation of how microglia function during postnatal development," but the results really do not provide any significant step forward. Based on the data presented, it may be that microglia phagocytosis only represents a clearance activity. Again, the authors state (p. 15) that they show "that ramified microglia are the dominant committers of phagocytosis in the developing cerebellum and this may be a component of the appropriate establishment of the cerebellar circuit." While this function of ramified is evident (as also shown in previous studies), the current study provides no firm basis for establishing the link between microglial phagocytosis and specific aspects of cerebellar circuit development or plasticity.

We appreciate the comments by reviewer #2. We recognize that this report is largely descriptive but note that the mission of eNeuro is to "publish excellent science that can be discussed, debated, studied and built upon". There is no requirement that studies be mechanistic or hypothesis driven, which is why we chose this particular journal. With regard to the scope of the work, we describe important and novel aspects of microglia during the development of the cerebellum allowing us to build a story based on the firm foundation of microglial phagocytosis. We believe this work sets the basis to formulate a hypothesis to test function or mechanism for the future by us or anyone in the field who finds this work helpful. We recognize that the results presented in this manuscript do not provide direct information regarding microglial function; therefore, we have modified our conclusion (page 2) and it now is stated in the manuscript as follows:

"This study provides important foundational profiles of microglial development in the cerebellum, a vulnerable structure to alteration during development, and contributes to the growing appreciation of the clearance activity of microglia during postnatal development".

We also agree that our study provides no firm basis for establishing a link between microglial phagocytosis and specific aspects of cerebellar circuit development or plasticity. Therefore, we have modified our conclusion in the text (page 15) as follows:

"Here we show that ramified microglia are the dominant committers of phagocytosis during the second and third postnatal week of development in the cerebellum. However, how phagocytic microglia contribute to the establishment of the cerebellar circuit remains poorly understood".

The authors' hypothesis is that "microglia change their morphological profile and cell number in direct relationship to the maturation of the cerebellum as well as the anatomical location within the cerebellar cortex." But given the current literature, what reasonable alternative hypotheses are there? Potentially, the observations could identify spatial-temporal patterns of activity of microglia phagocytosis of apoptotic cells or axon terminals/synapses, but the results suggest that neuronal cell death does not correlate well with microglial phagocytic activity (surmised from phagocytic cup frequency). This leaves open the significance of the observations. As such, the study does not significantly extend our understanding of the role of microglia in cerebellar (or brain) development.

Results in this study showed the peak of neuronal cell death does not correlate totally with peak of microglial phagocytosis in the developing cerebellum. Nevertheless, we showed that some phagocytic cups are engulfing dead cells. This suggests that something more complex is happening in terms of microglial phagocytosis in the developing cerebellum, especially when the phagocytic activity of microglia differs according to location in the cerebellar cortex, something that we here report for first time to the best of our knowledge. Thus, these data establish a platform for future studies addressing the role of microglial phagocytosis during the postnatal development of the cerebellum particularly in the granular layer.

Other specific issues:

1) The authors typically present a summary of their data before presenting the raw data. It is preferable, at least to this reader, to see the raw data before any summary or statistical analyses. For example, Fig. 1B provides a quantitative summary of the various morphological types of microglia, but examples of each morphological type are not presented until Fig. 2. The same is true for other analyses, including the phagocytic cups. Moreover, the authors should present representative examples of larger fields of view showing the range of morphological types of microglia, not just cropped images of isolated cells.

We want to clarify that we did not intend to present a summary of the data in the figures mentioned above. Instead, the order of the data are presented from the general (Fig. 1B) to the particular (Fig. 2) in order to highlight microglia features such as morphology, phagocytic activity and location. We have added new images in Figure 2 (panel A, B, C and D) representative of microglial morphology in larger fields as suggested.

2) Categorization of microglia is based on image analysis using the StereoInvestigator software. Although another study is referenced, the authors should state in the present manuscript how they defined "thick" and "thin" processes? It seems that this delineation could significantly alter the outcome. Moreover, the authors need to consider and discuss how any image processing may affect the morphometric analysis. For example, how does altering the brightness/contrast of the images affect the categorization of microglia with thick vs. thin processes? Could it be that phagocytic cups are more evident in microglia with "thin processes" and are rather obscured in the "thick processes" of microglia? Could this explain why there is a large spike in phagocytic cups at PN17, a time when microglia with "thin processes" begin to predominate?

We have stated in the manuscript our criteria to define microglia with thick and thin processes (page 7) as follows: "For descriptive purposes we identified microglia with thick and thin processes as follows: microglia with thick processes are generally large cells with an amorphous cell body, and with generally ramified short or long thick processes. Whilst, microglia with thin processes are large or small cells with a round and small cell body, with ramified short or long thin processes (more ramified than microglia with thick processes)".

With regards to the image processing and the morphometric analysis, we performed the morphometric analysis on the slide containing the cerebellar sections under the microscope keeping the camera and program parameters similar for each counting session. Thus, factors such as brightness did not affect our capability to identified microglial morphology or phagocytic cups. In addition, we identified and counted only those phagocytic cups located at the tip of microglial processes as shown in Figure 3B and 3C and stated on page 7. This way, identification and counting of phagocytic cups was clear and precise in either ramified microglia with thick or thin processes.

3) Related to this point, from the images shown it appears that microglia at PN12 have the thinnest processes (Fig. 3B), yet the quantitation in Fig. 1 indicate that microglia with thin processes predominate later in development.

The goal of Figure 3C, previously Figure 3B, was to depict the location of a phagocytic cup at the tip of a microglial process. The image the reviewer refers to coincided with a microglia with thin processes; nonetheless, it does not represent the most predominant microglial morphology at PN12. Therefore, in order to clarify this point we have changed it for another image with thick processes to be consistent with the entire image in panel 3C. Additionally, we have included more images of microglia with thick processes but without phagocytic cups in order to show both phagocytic and non-phagocytic microglia in the developing cerebellum.

4) The authors should describe the criteria they use for identifying "phagocytic cups." What is the lower limit in size of the structures they consider "cups"? Given that all of these phagocytic cups are expected to shrink and become completely resorbed during the degradation phase, what is the significance of the "average size" of the cups? As mentioned by the authors, the size of the largest cups suggests engulfment of fairly large structures such as cell bodies, rather than synaptic terminals. Small cups may simply represent shrunken larger cups. It would be helpful to indicate the absolute number of phagocytic cups counted, not just the average density. This could be done by showing a scatter plot of the individual cup sizes in addition to the mean values in Fig. 5D.

We have clarified in the text (page 7) our criteria for identifying phagocytic cups and it was stated as follows: "In order to have a clear identification of phagocytic cups, only those located at the tip of microglial processes with a round morphology (see Figure 3B-C) were counted".

For phagocytic cup size measurement, we did not consider a lower limit size of the cups as a parameter. As mentioned before, we identified visually the phagocytic cups based on their round morphology and their location at the tip of any microglial process, we measured their size and used that for statistical analysis. It is possible the size of phagocytic cups are influenced by the degradation phase, as suggested by the reviewer, but it is also possible the change is reflective of the formation phase of phagocytic cups. At this point, we do not know. As mentioned by the reviewer, small cups might represent shrunken larger cups, but also they may represent the engulfment of smaller structures such as basket or stellate interneurons in the molecular layer or the granule neurons or the glumerulus in the granular layer. Testing these possibilities will be important future work.

Lastly, we have changed the graph depicting the average size of phagocytic cups (Figure 5D) for a scatter plot depicting the individual cup sizes as well as the mean {plus minus} S.E.M. values.

5) The authors frequently use the term "number" when they may actually mean "density." E.g., p. 9, line 2, and the second bold heading on p. 10 ("more microglia" could refer to absolute number of microglia in a larger tissue volume or to higher cell density).

We have changed the term "number" for "density" in the manuscript as suggested.

6) The authors state (p. 17-18) that "sex is a factor that influences microglia in the cerebellum as males showed more microglia with thin processes than females..." Did the authors assess whether the size or weight of the animals was correlated with microglial density, or consider whether this could account for an apparent sex-link effect?

No, we did not assess the size or weight of the animals to correlate with microglial cell density as we did not find an overall sex difference, only a very few limited ones. Moreover, in our experience differences in weight and size between male and female rats become detectable only after they reach puberty and our study did not exceed 21 days postnatal.
